# News and Miscellany

**Published:** 1901-06

**Authors:** 


### News and Miscellany.

**The Commencement** exercises of Jefferson Medical College were held in the Philadelphia Academy of Music on May 15. There were 142 graduates.

**Texas** raised quarantine against San Francisco on 8th.

---

**This year's** graduating class at Tulane University numbered 125.

---

**Dr. R. L. McMeans,** formerly of Palestine, has moved to San Antonio.

---

**Reports from** Havana for March show the lowest death rate recorded since 1890, it being 26.28 per thousand.

---

**The South Texas Medical Association** will meet in Houston June 20-21. Don't forget it. Rates as usual.

---

**This year there** are eleven students from Texas matriculated in the Medical Department of the University of Pennsylvania.

---

**The Louisiana** State Board of Medical Examiners reported seventy successful applicants for license among ninety-five who appeared for examination.

---

**At a recent** meeting of the Mississippi State Board of Health ninety-seven out of one hundred and twenty-seven applicants were granted license to practice medicine.

---

**Briggs** of the B. V. and Guinn of the E. T. M. C. Society, are both rattling good secretaries. Re-elected for life,—that is for "en durin the war." Briggs was a confederate surgeon.

---

**San Francisco's** Chinatown has at last received a thorough cleaning and disinfection, and the State has appropriated $25,000 to assist the city authorities in keeping it in good sanitary condition.

---

**A Doctor's Union.**—It has been proposed that the doctors organize themselves into a union, and that the undertakers be requested to cooperate by refusing to accept for burial a body not killed by a union doctor.

---

**A Brazos Bottom Doctor** at the Calvert meeting gave me his subscription and address. I said, "Doctor, what sort of a place is Stone City?" "No city at all," said he; "it is only a bad place in the road, worse than the other places."

---

**Married** at Catonsville, Md., June 4 inst., Dr. Hugh Hampton Young, of San Antonio, Texas, now of the Johns-Hopkins University, Baltimore, to Miss Bessy Mason Colston, daughter of Mr. and Mrs. Frederick M. Colston, of Catonsville.

**I want a** few copies of the March (1901) number of the "Red Back." I will pay 10 cts. each for them; or will advance subscription one month for each copy, or will send a copy of the New Medical Law of Texas—"pamphlet form, with remarks"—in red cover for each copy.

---

**This Issue** completes Volume Sixteen of the "Red Back," and still she booms. It will begin Volume Seventeen with a greatly increased subscription list, and the largest, cleanest and best paying advertising patronage anywhere. Send in your subscription doctors, and keep up with the procession.

---

**A Suggestion.**—If Beaumont would utilize the output of two or three of its "gushers" to keep a coating of oil on the filthy and stagnant water in the neighborhood and thus prevent the development of a few billion mosquito larvæ, its business men and visitors would suffer less from having their oil fever complicated with malaria.

---

**The work** of repairing the damages to the building of the Medical Department of the University at Galveston done by the storm, September 8th, 1900, will be inaugurated at once, appropriations for that purpose having been made by the 27th Legislature. The Medical Department will hold its Commencement exercises June 15th.

---

**The Tuberculosis Congress** held in New York May 15, 16 and 17 (ult.) under the auspices of the Medico-Legal Society was a gratifying success, and a most important gathering. Hon. Clark Bell, the founder of the society, delivered the opening address. The address, together with a full report of the proceedings, will appear in the June number of the *Medico-Legal Journal.*

---

**San Antonio's** City Health Officer, Dr. F. Paschal, reports a decrease of 50 per cent in the mortality from typhoid fever for the year ending May 31, 1901, as compared with the average mortality from this disease during the past ten years. This is the result of the introduction of a fine system of sewers, and a thorough policing of all premises under the vigilant supervision of a zealous and enlightened health officer.

---

**Texas Boys at Nashville.**—We notice amongst the graduates of the Medical Department, University of Nashville, session just closed, the following named Texas boys. J. J. Blanton, A. B. Moody, K. W. Rowe. And amongst the graduates of the Medical Department of Vanderbilt University the following: J. H. Carraway, C. C. Collom, J. I. Bearson, W. O. Stephenson, G. W. Stone, J. F. Taylor and J. Ward, Jr.

**Dr. Deering J. Roberts,** the well known old Confederate surgeon and editor of the *Southern Practitioner* (Nashville), (the official organ of the Association of Confederate Surgeons, a publication now in its 23rd year,) who was born and raised in Nashville, was recently elected president of the Tennessee State Medical Association unanimously, by rising vote, by acclamation. A higher testimonial of appreciation of a man's life labors could not be bestowed on any one. We feel proud of his election.

---

**At the recently** adjourned session of the Legislature, $10,000 was appropriated for a mineral survey of the State to be conducted under the supervision of the Regents of the University of Texas. This survey has now been organized with Dr. Wm. B. Phillips at its head. Dr. Phillips is Professor of Economic and Field Geology and Mineralogy in the University of Texas. Dr. Ben. F. Hill will be field assistant, while the work of chemical analyses will be done by Dr. H. W. Harper, with O. H. Palm and S. H. Worrell as assistants. The work of this survey is to determine the mineral resources of the public lands of Texas. Analyses of specimens submitted by any citizen of the State will be made in the Chemical Laboratory in Austin at fees fixed by those who have the survey in charge.

---

### Experiments with Disinfectants.

---

Dr. John J. Archinard, having conducted a number of experiments to test the efficacy of the various disinfectants in common use, draws the following conclusions:

1. Sulphur dioxide being a disinfectant of low efficiency and worse still a destroyer of dyes, whilst utterly impracticable for thorough application, should be discarded at once.

2. Formaldehyde, on the other hand, is an agent of a great penetration and high disinfectant efficiency, though practically harmless to colors and fabrics. Apparatuses are now being made for disinfecting rooms and contents by means of simple, cheap, harmless and easily managed methods of rapidly generating this gas. Therefore, the immediate adoption of formaldehyde is much to be desired.

3. Bi-chloride of mercury recommends itself as a handy and efficient means of treating washable fabrics.

4. Sprinkling with twenty per cent. solution of formalin and keeping in close containers for twenty-four hours, enables us to dispose of woolen and other wearing apparels not amenable to treatment by method third.

5. Chlorinated lime is our best method for disinfection of excreta, privy vaults, yards, drains, etc.

6. Incineration is the best method of disposing of substances of no intrinsic value, such as rags containing dejecta, etc.—*New Orleans Medical and Surgical Journal,* May, 1901.

### The Treatment of Compound Fractures.

In discussing the treatment of compound fractures of the long bones which are not associated with too much injury to the soft tissues, Dr. D. C. Moriarta objects to the practice in vouge with most physicians of attempting to replace the ends of the fractured bones by extension, the sealing up of the wound, and the putting on of a retaining splint. He thinks that, while good results are obtained in this way, the most careful surgeons are apt to get a certain per cent. of avoidable bad results.

His plan of treatment is to make a free and deep incision, exposing the fractured ends of the bone. He then cleans the wound, cares for the torn tissues, removes all foreign substances and approximates accurately the fractured ends and holds them in position by wiring or nailing. In every case of oblique fracture he wires the ends with heavy silver wire.

By pursuing this course of treatment he removes all danger from infectious material that may have been introduced through the wound, and from the bruised tissues and loose spicules of bone.

In conclusion, he says: "I earnestly urge that in compound fractures, when the pathological condition is not determined, that the puncture or laceration be enlarged, the bone fully exposed, approximated and held there, if you are sure of your technic and the patient's condition does not contraindicate it. I am sure that the dangers of such procedure are nil, while its advantages may save a life or limb; that the results are nearer perfect and quicker; and many unsatisfactory secondary operations may be avoided."—*American Medicine*, May 4, 1901.

